# Acceptability of *Aedes aegypti* blood feeding on dengue virus-infected human volunteers for vector competence studies in Iquitos, Peru

**DOI:** 10.1371/journal.pntd.0007090

**Published:** 2019-02-11

**Authors:** Amy C. Morrison, Julia Schwarz, Kanya C. Long, Jhonny Cordova, Jennifer E. Rios, W. Lorena Quiroz, S. Alfonso Vizcarra, Robert D. Hontz, Thomas W. Scott, Louis Lambrechts, Valerie A. Paz Soldan

**Affiliations:** 1 Department of Entomology and Nematology, University of California, Davis, Davis, California, United States of America; 2 Virology and Emerging Infections Department, U.S. Naval Medical Research Unit No. 6, Washington DC, Lima and Iquitos, Peru; 3 Icahn School of Medicine at Mount Sinai, New York, New York, United States of America; 4 Insect-Virus Interactions Group, Department of Genomes and Genetics, Institut Pasteur, Paris, France; 5 Centre National de la Recherche Scientifique, Unité de Recherche Associée 3012, Paris, France; 6 Global Community Health and Behavioral Sciences, Tulane University School of Public Health and Tropical Medicine, New Orleans, Louisiana, United States of America; International Centre for Genetic Engineering and Biotechnology, INDIA

## Abstract

As part of a study to investigate drivers of dengue virus (DENV) transmission dynamics, this qualitative study explored whether DENV-infected residents of Iquitos, Peru, considered it acceptable (1) to participate in direct mosquito feeding experiments (lab-reared *Aedes aegypti* mosquitoes fed directly on human volunteers) and (2) to provide blood meals indirectly (*Ae*. *aegypti* fed on blood drawn from participants by venipuncture). Twelve focus group discussions (FGDs; 94 participants: 82 females and 12 males) were conducted in January 2014 to explore six themes: (1) concerns and preferences regarding direct mosquito feeds and blood draws, (2) comprehension of and misconceptions about study procedures, (3) motivating factors for participation, (4) acceptability of children’s participation, (5) willingness to provide multiple samples over several days, and (6) preference for direct feedings in homes versus the study laboratory. Results of FGDs, including one with 5 of 53 past direct mosquito feed participants, indicated that mosquito feeding procedures are acceptable to Iquitos residents when they are provided with information and a few key messages are properly reinforced. FGD participants’ concerns focused primarily on safety issues rather than discomfort associated with mosquito bites. A video explaining the study dramatically increased comprehension of the study procedures. The majority of participants expressed a preference for mosquito feeding over venipuncture. Adults supported child participation if the children themselves assented. For most participants, home feedings were preferred over those in a laboratory. A major impetus for participation was the idea that results would contribute to an improved understanding of DENV transmission in Iquitos. Findings from our study will support future large-scale studies that employ direct mosquito feeding, a low-risk, non-invasive procedure that is experimentally superior to artificial mosquito feeding methods.

## Author summary

Approximately half of the world’s population is at risk of contracting dengue virus (DENV). Ethical and logistical concerns with feeding lab-raised mosquitoes directly on naturally infected human subjects, and the lack of a relevant animal model for DENV experimental infection, are important obstacles to better understanding DENV transmission from humans to mosquitoes. Results from artificial infectious blood meals can bias estimates of mosquito infection and transmission rates. Based on 12 focus group discussions, we determined that the practice of feeding uninfected lab-raised mosquitoes on naturally infected human subjects is highly acceptable to people living in Iquitos, Peru, especially after common concerns are addressed. The majority of participants were willing to have mosquitoes feed on them directly and to give venous blood samples to feed to mosquitoes indirectly. Most participants stated a preference for direct feeding. This formative research, including recognition of and addressing common misconceptions, will help guide future development of protocols using biologically relevant direct mosquito feeding methods.

## Introduction

Vector competence studies on the intrinsic ability of mosquitoes to transmit human pathogens have increased in importance and frequency with the emergence of epidemic *Aedes aegypti*-borne viruses such as dengue (DENV), chikungunya (CHIKV), Zika (ZIKV), and yellow fever (YFV) viruses [1]. Historical methods to determine vector competence had important experimental constraints that limited researchers’ ability to extrapolate to natural transmission and to understand the significance of data from prospective epidemiological studies for humans.

Limitations of laboratory-based vector competence studies include using artificial infectious blood meals that are not the same as feeding directly on a human host and laboratory-reared mosquitoes from colonies that are many generations removed from field populations and do not represent vector competence of wild mosquitoes [2]. Artificial infectious blood meals, often composed of cultured virus mixed with animal blood and presented to mosquitoes across a skin-simulating membrane, use virus passaged in cell culture, which can select for viruses that are not maintained in nature [3], altering the ability of a virus to infect and/or replicate in a mosquito [4]. The common use of defibrinated blood in artificial blood meals can similarly change the distribution of virus in a mosquito midgut, resulting in a systematic misrepresentation of experimentally exposed mosquito infection rates [5].

The most realistic way to overcome these limitations is to allow uninfected mosquitoes to take a blood meal directly from a naturally infected human volunteer or, alternatively, to imbibe blood from an artificial feeding apparatus that was drawn from an infected person. These kinds of experiments present logistical and ethical challenges. We know of no published recommendations to guide Institutional Review Boards (IRBs) in evaluating the use of human participants in these procedures [6].

Researchers in several studies explored interactions between malaria parasites (*Plasmodium* spp.) and anopheline mosquitoes by feeding laboratory-reared mosquitoes on people with active malaria infections, alone or in comparison with indirect feeding (infectious blood drawn from a participant and delivered by artificial feeder) [7–9]. The fact that treatment is available for malaria makes these procedures more acceptable to IRBs than when treatment is not available, as is the case for arboviral diseases.

Since publication of the Belmont Report in 1978 [10], which marked the initiation of IRB review of human use protocols, we are aware of only three research groups that have carried out experiments of direct mosquito feeding on human subjects naturally infected with DENV [11–13]. All of these were conducted in Southeast Asia. In Vietnam, 407 direct mosquito feedings were carried out on 208 hospitalized dengue patients, ranging from 19–30 years of age, with no adverse events reported [12]. In Singapore, direct feeding was completed on 26 hospitalized adults [13]. In Cambodia, 164 direct mosquito feedings were carried on household contacts of known dengue cases. These participants had not received laboratory results about their infection status at the time of feeding; 89% of the participants were less than 16 years of age [11].

In late 2010, our research group initiated the process to obtain IRB approval for a pilot study designed to compare direct with indirect mosquito feeding methods using blood from naturally infected study subjects recruited from our ongoing community- and clinic-based febrile surveillance protocols. Our initial objective was to obtain preliminary data for a large-scale research program that would resolve the long-standing enigma about the contribution of people with inapparent and mild symptomatic infections to DENV transmission dynamics. We aimed to determine whether indirect methods could be used in lieu of direct mosquito feeding experiments. Approval to carry out our study was granted in May 2011 with multiple IRB stipulations, including close monitoring [34]. We enrolled our first participants during September 2012, and after a year of participant interactions realized that direct mosquito feedings were potentially acceptable on a large scale. As we continued to enroll participants in the companion vector competence study, we requested IRB permission to (1) expand inclusion criteria to younger participants, (2) move mosquito feedings from the laboratory to participants’ homes, and (3) conduct focus group discussions (FGDs) on the acceptability of direct mosquito feeding experiments.

Here, we present results from 12 FGDs conducted during January 2014. Our goals were to (1) identify community concerns and misconceptions associated with the direct mosquito feeding procedures, (2) identify key messages to ensure comprehension of our study, (3) assess willingness to participate in study protocols requiring direct mosquito feeds and multiple blood draws over the course of a single dengue infection, (4) determine the acceptability of allowing young children to participate in mosquito feeding experiments, and (5) determine the acceptability of conducting direct mosquito feedings in the participants home environment rather than the study laboratory. Our long-term objective was to use community-derived opinions to inform subsequent IRB applications and to finalize protocols for planned, follow-up larger-scale vector competence studies.

## Methods and materials

### Ethical considerations and consent

The study protocol was approved by the U.S. Naval Medical Research Unit No. 6 (Protocol #NAMRU6.2011.0002) Institutional Review Board, which includes Peruvian representation and complies with US Federal and Peruvian regulations governing the protection of human subjects. IRB authorization agreements were established between U.S. Naval Medical Research Unit No. 6, University of California, Davis, and Institute Pasteur. The protocol was reviewed and approved by the Loreto Regional Health Department (LRHD), which oversees health research in Iquitos. Consent without written documentation (verbal) was approved by the NAMRU-6 IRB so that no names were recorded or stored by the investigator.

### Study setting

This study took place in Iquitos, Peru, located in the northeastern Amazon basin, where the human population is approaching 400,000 inhabitants [14] and divided into four districts: Maynas, Punchana, Belen, and San Juan Bautista. Detailed descriptions of the city, its *Aedes aegypti* population, and local DENV transmission have been published elsewhere [15–23]. This region is geographically isolated; it can only be reached by boat or airplane. The main industries in this region are small commercial entreprises, and of extractive (logging, mining) or agricultural nature. Iquitos has experienced rapid urbanization in the past three decades [14], from the neighborhoods around the city center/commercial zones in the districts of Maynas and Punchana to areas on the river (Belen and parts of Punchana) and to the South (San Juan Bautista).

The recruitment neighborhoods described in the next section were located in the more developed and central neighborhoods of Maynas and Punchana, which are relatively homogenous, with a patchwork of households ranging from wood structures with dirt floors to brick and concrete and ceramic floors. The Peruvian Statistics and Information Institute states that about 30% of the urban jungle population of Peru has at least one unmet basic need, 18.2% live in poverty, and 3% of the population in extreme poverty [24]. Evidence of extreme wealth is not observed in Iquitos, and luxury items such as air conditioners are rarely observed. Other indicators for the Maynas and Punchana districts include that 93% of structures are individual houses (row houses with shared walls), 82–38% have corregated metal roofs, 90–97% have electricity, and literacy rates are 88–92% [25].

### Study subjects

Purposive sampling, a non-probabilistic sampling method commonly used in qualitative research [26], was used to recruit focus group participants from neighborhoods in the Amazonian city of Iquitos, Peru. We carried out 12 FGDs with a total of 94 residents over a single week during January 2014. To facilitate recruitment and transportation of individuals to the NAMRU-6 conference room, 2–3 residents per block within 5–6 contiguous blocks were recruited in person, door-to-door, 2–3 hours before initiation of each FGD (for a total of 7 FGDs and 49 participants) from three neighborhoods where prospective cohort studies have been ongoing since 2007 [19, 22, 27], and two neighborhoods (for a total of 4 FGDs and 40 participants) where no dengue studies have been conducted by our group since 2007. All five neighborhoods were located in the districts of Maynas and Punchana in the center of the city, representing more developed and economically stable neighborhoods than observed in neighborhoods located in the far north and south and river edges of the city. The neighborhoods without ongoing research studies were either next to or within a few kilometers of the neighborhoods with ongoing research. For context, neighborhoods with ongoing prospective studies were visited by our staff approximately three times per week to identify people with febrile illness. Our experience in Iquitos is that women usually make the health-related decisions for the family, including children, hence we intended to have a larger representation of women when requesting household participation in FGDs. One FGD was conducted with previous mosquito feeding participants (n = 3) and their mothers if the participants were minors (n = 2). At this time, the Asian-American genotype of DENV serotype 2 was circulating and Zika virus had not been detected in the city [28].

### Focus group methodology

A Peruvian social scientist with over a decade of work experience conducting FGDs in Iquitos (VAPS) facilitated the FGDs in Spanish. A Spanish-speaking expert in the mosquito feeding procedures (ACM) was present in all of the FGDs to answer technical questions. Two research team members took detailed notes of the discussion and two more assisted in recording ideas and thoughts on large sheets of paper (that all could see) and in role-play exercises. Before each FGD, an IRB-approved consent form was read and participants and parents of minor participants provided verbal consent to participate in the study and be audiotaped.

**Fig 1:**
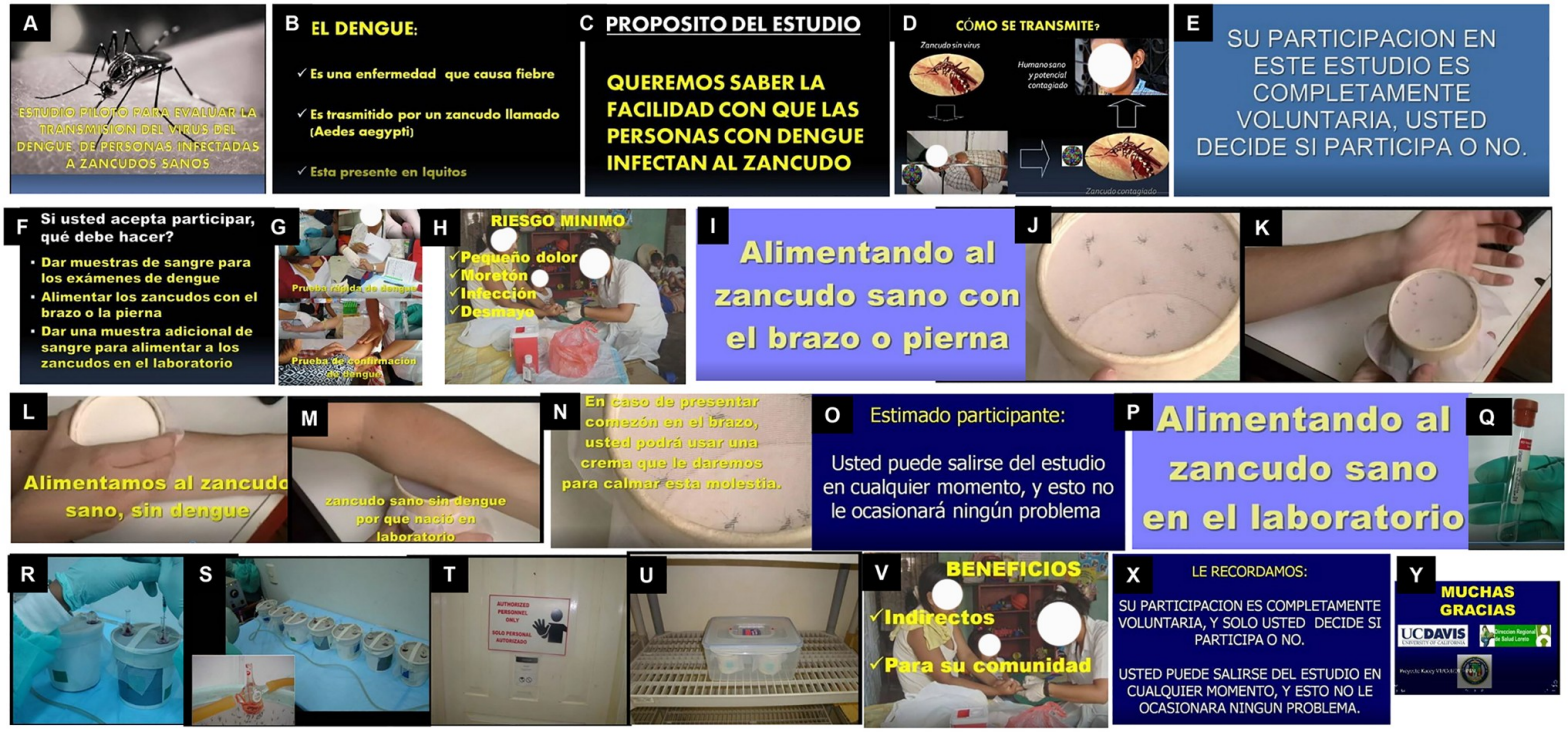
Key screen shots from mosquito feeding consent video. (A) Project Title. Pilot study to evaluate the transmission of dengue virus from infected people to healthy mosquitoes. (B) Simple information on dengue. (C) Study objective. We want to know how easily people with dengue can infect mosquitoes. (D) Dengue transmission cycle. (E) Disclaimer. “Your participation is completely voluntary, you decide to participate or not. (F) Participation included a blood sample to findout if you have dengue, feeding mosquitoes with your arm or leg, and given more blood when you feed the mosquitoes. (G) Taking blood samples. (H) Risks. pain, bruise, infection, fainting. (I) Feeding healthy mosquitoes. (J-M) Feeding healthy mosquitoes, without dengue that were grown in the laboratory. (N) Providing itch cream if needed. (O) Disclaimer, (P-S) Feeding mosquitoes in the laboratory. (T-U) Storing the mosquitoes safely. (V) Study benefits. (X) Disclaimer. (Y) Thank you.

We developed and applied an FGD guide to ensure we covered the same topics in each FGD. Each FGD began with a brief introduction and an explanation that we wanted to learn more about dengue and how DENV is transmitted from infected people to mosquitoes, followed by a brief description of the direct and indirect mosquito feeding methods. Role-playing exercises were used to simulate an invitation to participate in the project. Initially, we also intended to evaluate a mosquito feeding consent video that we had developed for the pilot project (see description below, S1 Video, Fig 1), but, after our first two FGDs in which the video was shown at the end for discussion and feedback, we decided to show this video immediately after the brief introduction because it became apparent that it was an effective tool for introducing the project and procedures to the group. This also mirrored how the study was presented to potential participants in the field. To assess people’s understanding of the direct mosquito feeding procedures, after the brief discussion and viewing of the consent video, we asked participants to describe the purpose of this study, the procedures, and their initial reactions. To ensure that these hypothetical questions felt real to the FGD participants, one member of our research team (ACM) had mosquitoes feeding on her during the FGD to show them what the mosquitoes in a container looked like, and what her legs looked like post-feeding. We noted the types of questions and discussion among the participants. We wanted to assess how well people understood the purpose and procedures, identify concerns participants had about the process, assess whether they would participate or allow their children to do so, and identify any additional concerns regarding their children’s participation. Groups were asked to state their preferences for providing a venous blood sample to feed to mosquitoes indirectly or feeding the mosquitoes directly on their arms or legs, and discuss the pros and cons of each method. Individuals were asked about how many consecutive days they would be willing to provide both venous blood samples and directly feed mosquitoes.

Regarding the mosquito feeding consent video, our primary objective was to clearly show the mosquito feeding procedures to potential participants. We wanted people to see a full cup of mosquitoes biting an arm and becoming engorged with blood. The video includes the following: (1) the title and purpose of the project, (2) a brief description of the DENV transmission cycle and how one can become infected, (3) an explanation of what is expected from a participant in the project (mosquito feeding and provision of blood samples), (4) the study’s risks and benefits, (5) a visual of mosquito feeding procedures (i.e., mosquitoes feeding on an arm and mosquitoes feeding on blood through an artificial feeder), (6) clear statements that the mosquitoes used in the experiments were laboratory reared and laboratory tested to be free of DENV, and (7) an explanation that after the mosquitoes are fed they are held in a secure laboratory environment.

### Experiences of study participants with direct mosquito feeds

Between September 2012 and January 2015, we enrolled 58 DENV-positive subjects from a total of 197 people (50% female) who agreed to participate in direct mosquito feeds if they were identified as having an active DENV infection [34], representing about 70% of the febrile people invited to participate in the study. Of these, 53 subjects (35 males, 18 females) participated in direct mosquito feeds. The remaining five (4 females, 1 male) agreed to only participate in indirect feeds (drawing their blood and artificially feeding it to mosquitoes). During and directly after the procedure, we asked participants how they felt about doing the feeds, whether they wanted to continue, and if they would do it again.

### Data management and analysis

After the FGDs, the entire team present during FGDs compiled all notes to produce a detailed report of each session and discussions that took place. Audiotapes were not transcribed, but were used to fill in gaps in notes and to obtain exact quotes. Data was segregated by themes in the focus group guide: (1) comprehension of project, based on misconceptions expressed by participants, (2) questions and concerns about direct and indirect mosquito feeds, (3) willingness to allow children to participate, (4) number of times individuals would be willing to participate in daily indirect and direct mosquito feeds, and (5) preference for participation in their home versus in the laboratory. Codes were developed based on these themes, as well as subthemes, by the two lead investigators (VAPS, ACM). Data was further stratified by participant—those from surveillance and non-surveillance areas. All notes were coded by one member of our research team who was not present during the FGDs, using Dedoose qualitative analysis software, version D.7.5.16. Additional sub-themes that emerged during coding were discussed and added if appropriate, and the person coding returned to previous transcripts to ensure all sub-themes were included. Any questions in the coding process were resolved by discussions between the two lead investigators present at FGDs, along with the person coding. Results are presented based on these main themes.

## Results

Each FGD had 6–10 participants; by design, the participants were predominantly female, and their ages ranged from 18 to 71 years (Table 1). Overall, more concerns were articulated in earlier than in later FGDs, and many of these concerns became less pronounced as study comprehension increased through a complete viewing of the consent video early in the FGD (versus near the end, as described in the study design section above.) We began with results from the 11 FGDs with individuals who did not participated in mosquito feedings, and then proceeded to the one FGD with participants from previous direct mosquito feeding experiments.

**Table 1:** Composition of 12 focus group discussions examining the acceptability of direct mosquito feeding experiments carried out on DENV-infected people.

FGD No.	No. Participants				Age: Range and Mean	Cohort
	Total # of participants	Women	Men	Participants with children <10 years of age		
1	9	5	4	4	33–71 (NA*)	surveillance
2	9	6	3	7	27–70 (NA)	surveillance
3	6	6	0	3	40–73 (NA)	surveillance
4	8	6	2	8	18–64 (NA)	surveillance
5	8	8	0	8	23–40 (32)	surveillance
6	9	9	0	6	30–48 (33)	surveillance
7	10	10	0	8	27–45 (29)	surveillance
8	8	8	0	10	26–54 (41)	non-surveillance
9	6	5	1	5	33–52 (43)	non-surveillance
10	8	8	0	7	20–44 (33)	non-surveillance
11	8	8	0	10	29–42 (37)	non-surveillance
12	5	3	2	2	19, 31, 45**	Participants in pilot blood feed
TOTAL	94	82	12	78	18–73	

*In the first four FGD, we only captured the age range in our notes and can not calculate the mean.

**Two individuals in this FGD were parents of children who had dengue and accepted a pilot mosquito blood feed. We did not obtain the age of the parents who participated in the FGD, only the age of their children: one was 10, the other was 15.

### Participant concerns

#### Safety

In all FGDs, except those conducted with previous mosquito feeding participants, a principal concern was the safety of the study (Table 2). This concern was primarily about the infectious status of the mosquitoes used in the experiments. Participants asked if the mosquitoes were “*healthy”*, “*infectious”*, “*sick”*, or “*infected”*. Some expressed fears of contracting dengue through the mosquitoes and wanted to know how researchers could verify that the mosquitoes used in the experiments were safe or *“clean”* and, in one case, how researchers could “*guarantee that the mosquitoes that fed on infected people would not go on to infect more people*.” These were important concerns expressed in 9 of 11 FGDs. In early FGDs, the number of people expressing this concern was higher than in later FGDs, likely because the consent video was played at the end of earlier FGDs. There was at least one participant in 9 of 11 FGD who asked if the mosquitoes were infected. In one FGD, participants suggested they would be more convinced if the research team fed the mosquitoes on themselves. One team member (ACM) demonstrated the feeding on herself during each FGD, and this led to a discussion about how we could know that the mosquitoes were clean. Other participants, especially those who understood the message that mosquitoes were clean and came from the laboratory expressed their faith in the research team, stating “*we trust you*,” “*take you at your word*,” “*it would be crazy to think you were doing something bad*,” and similar sentiments. Overall, the safety concern dissipated with more information and better comprehension of the study design.

**Table 2:** How key misconceptions identified in focus group discussions translated to changes in research protocol for direct mosquito feeding experiments carried out on DENV-infected people.

Key Misconceptions	# Times Mentioned in FGD in FS Zones (7)	# Times Mentioned in FGD in NS Zones (4)	Key Quotes	Actions
Study Safety	6	3	“Son los zancudos sanos o enfermos?” (Are the mosquitoes healthy or sick?) “Esos son zancudos sin dengue?” (Those mosquitoes do not have dengue?) “No confío que están sanos” (Don’t trust that the mosquito is truly safe) “Tal vez dentro de ese grupo hay un zancudo enfermo” (There might be one mosquito in the group that is sick)	During the consent process, we ensured it is clear that mosquitoes are used one time for feeding experiments and then are confined to the laboratory until they are killed and tested for dengue. Overall, we had the most success when we used the following language: “We take blood samples to study your dengue on the inside, check your temperature and symptoms to see how you are on the outside, and feed mosquitoes to see how they are affected.” This is the language currently used in the consent process.
Reaction to Mosquito Bite	5	2	“Da comezón, te deja bolitas” (It will itch and leave welts) “Me hacía herida como globitos y se infectaba. Ahora ya no” (Bites used to cause a small wound and welt and they got infected, but not anymore)	Our team took alcohol packs and Itch (Betamethsone 5%) cream that was left in participant’s home as requested.
Time Needed for Procedure	4	0	“Es más rápido” (It is faster [to get blood drawn than participate in the mosquito feeds experiment]).	IRB approval was obtained to carry out experiments in homes to reduce time burden.

A key idea that needed reinforcement multiple times was that mosquito feeds would only be carried out on people who had laboratory confirmation of a DENV infection; i.e., only on people who were already infected with DENV. Participants asked questions like “*so the person would already have dengue*?” or *“can it be anybody or [only] someone who has dengue*?*”* Once participants understood that we were studying DENV in people with an existing infection and not trying to infect people with DENV in order to study the disease, most individuals expressed relief that we were not conducting feeds on uninfected individuals. This helped us understand points that needed to be very clearly stated to potential participants. Another important point that required clarification was the use of different mosquitoes (a new cup of mosquitoes) for each participant; i.e., the same cup of mosquitoes would not be used again on another person. Participants in one FGD suggested that we add a statement in our consent process that mosquitoes are used only one time for feeding experiments and then are confined to the laboratory until they are killed and tested for DENV.

One group asked to see where we held the mosquitoes and wanted to know more details on how we knew they were clean. We showed them our insectary facility. ACM explained the 3-step process used to rear mosquitoes used in feeding experiments and how we determined they were not infected with DENV. First, eggs were collected from houses in Iquitos, brought to the laboratory, hatched, and raised to adulthood; these are the grandparents. Eggs from the grandparents were collected, hatched, and raised to adulthood; these are parents of the mosquitoes that feed on people. Third, eggs from the parents were collected, hatched, and raised to adulthood; these are the mosquitoes used in feeding experiments. We explained that all “mothers” were all tested for DENV to make sure they were uninfected or clean. Because the experimental mosquitoes could not have been exposed to the virus outside of the laboratory and could not become infected from their uninfected mothers, the experimental mosquitoes were free of DENV infection. The FG participants that we reviewed this process with recommended that we make a separate video and/or pamphlet explaining details of rearing clean mosquitoes for experimental feeds.

#### Discomfort from mosquito bites

A few people from most of the FGDs expressed the idea that they would feel discomfort from physical reactions (e.g., welts, itching) to the mosquito bites. Most groups indicated this was a problem only for people who are sensitive to bites and that many people were accustomed to being bitten and no longer showed reactions (Table 2). Participants in one group explained that the physical reactions could be a problem for infants and toddlers. Unexpectedly, most participants showed minimal concern for this issue and made clear statements to that effect, i.e., they already had a heavy bite burden in their homes, they were used to being bitten, and any effects from bites went away quickly or could be easily controlled with cream or alcohol.

#### Being experimental subjects

Concern about being an experimental subject was not widespread. In 2 of the 4 FGDs with participants who had not had little or no previous exposure to our studies, a few participants expressed concern with being part of a scientific investigation. Most notable was the comment that “*nobody wants someone to experiment with their bodies*,” or comments stated with a negative tone about “*being food for mosquitoes*,” or “*really*, *my arm or leg will contribute to an investigation*?” These attitudes seemed to dissipate after discussion of their concerns.

### Comprehension of study purpose and mosquito feeding procedures

We determined that the most effective way to communicate the purpose and procedures of the mosquito feeding experiments was to early in the FGD present the video (Fig 1, S1 Video) used in the informed consent process. We decided that the video should be preceded by a brief explanation of its content and why it was important. Based on the types of questions answered after the video, it was clear that many participants required further explanation, interaction, and reinforcement of key points by the research team.

To test comprehension, we asked participants “why are we doing the study?” Initial responses varied widely, but the most common responses indicated that participants captured two key concepts: (1) we wanted to know more about dengue, and (2) we were trying to understand what would happen to the mosquitoes after they fed on an infected person. Participants used phrases like “*you want to see what kind of reaction the mosquitoes will have*” or *“[you want to see] if we can give the mosquitoes dengue*.” Although it was clear that participants in many cases tried to repeat messages from the consent video, their focus on transmission from humans to mosquitoes demonstrated an understanding of why feeding mosquitoes blood, either directly or indirectly, was an essential part of the project. Comprehension of this key message was incomplete initially, with some participants expressing interesting misconceptions (see below and Table 3). In most groups, however, individuals who grasped the concept that we were interested in what happened to mosquitoes, rather than what happened to human participants, provided explanations to others in the group who did not understand this concept. This method of peer-to-peer explanation helped researchers assess how well some participants had understood, as well as allowed us to document how the concept was expressed among the participants themselves so we could use their language in information materials developed in the future.

**Table 3:** Assessment of comprehension of participants in 11 focus group discussions about the reason the mosquito feeding studies are being carried out and to verify that the objectives stated in the video were clear.

Themes that emerged: *We are doing these studies…*	Quotes
To learn more about dengue	“Saber más sobre el dengue” (Know more about dengue) “Para poder prevenir” (To be able to prevent) “Participaría para saber más acerca del dengue” (You would participate to know more about dengue)
To study transmission to mosquitoes	“Saber qué reacción tendrían los zancudos” (Know what reaction the mosquitoes would have) “Queremos ver si le contagiamos al zancudo” (We want to see if we can give the mosquitoes dengue) “Transmisión al zancudo” (Transmission to the mosquito) “El zancudo se va a contagiar de dengue (The mosquito is going to catch dengue) “Como los zancudos reaccionarán al dengue” (How the mosquito will react to dengue) “El comportamiento del virus en el zancudo” (The behavior of the virus in the mosquito)
Because it is a novel way to diagnose dengue *	“Es para averiguar si tenemos dengue” (It is to find out if we have dengue) “Para confirmar que estamos con dengue” (To confirm if we have dengue) “Los zancudos nos muerden para ver si tenemos dengue” (The mosquitoes bite us to see if we are infected with dengue) *“Yo si*, *para saber con que tipo de dengue estoy contaminada” (I would do it to find out what kind of dengue I’m infected with)*
Because we are studying the reaction of people to mosquito bites *	*“Cuáles son los síntomas después de un zancudo muerde*?*” (What are the symptoms after the mosquito bites*?*)* “Descubra lo que un zancudo puede hacerle a ti” (Find out what the mosquito can do to you)
Because we are using the mosquito feeding to monitor a person’s illness *	“*Entendí para analizar cuál de los zancudos da dengue*, *creo que da dengue es el que da comezón*” (*One particularly interesting comment was a person who thought the amount of itching was correlated to the amount of dengue*).
Because mosquito feeding has beneficial effects *	“Porque alimentar zancudos reducirá la cantidad de virus en mi sangre” (Because feeding mosquitoes will reduce the amount of virus in my blood)

* These are misconceptions expressed by FGD participants. It was important to understand the most common misconceptions to try to avoid them in the future by reinforcing information about these topics.

#### Misconceptions

The most important misconception was that our proposed experiments were intended to infect people, rather than to infect mosquitoes by feeding them on people when they had a DENV infection. Participants required clarification and verification, sometimes multiple times, that the experiments were not using mosquitoes infected with DENV. Many participants captured this message easily, especially those who viewed the consent video early in the FGD. Others needed further clarification. For example, one participant explained that “*the video showed us that they [the mosquitoes] are not contaminated with the disease*,” in response to others who asked, “*so these mosquitoes don’t have dengue*?”

Another misconception expressed by some participants was that the purpose of the study was to determine whether they, the human participant, had dengue. The video did state this as an objective, indicating that we would determine whether they had dengue by testing their blood. This was technically correct, but some of FGD participants interpreted the feeding experiments as a unique way to diagnose their illness. They saw this as a form of xenodiagnosis, although this term or concept was not specifically discussed. Related was the belief by some participants that the experiment would monitor their illness through the mosquito feeds (“*we will find out when we don’t have dengue anymore*”) or that the feeding would reduce the amount of virus in their blood and therefore be beneficial to them. There were a few comments about participants being food for mosquitoes. One FDG participant stated that we were feeding mosquitoes on blood so that we could grow more mosquitoes.

One limitation to comprehension was age. We found that some members of our population, especially those over 60 years of age, had a difficult time providing responses to the hypothetical scenario of being asked to participate in a research study or to feed mosquitoes. Some repeated their responses of “but I don’t have dengue” to our hypothetical scenarios.

Misconceptions were addressed at the end of each FGD, and, by then, the majority of participants understood the purpose of the project and acknowledged that it was their initial “*failing to understand that had left them with doubts and concerns*.” After they understood, they stated they would be comfortable participating in direct mosquito feeding, and the research team noted what points needed more emphasis and clarification to help people understand (Table 2).

### Motivation to participate in direct mosquito feeding experiments

When asked what would motivate them to participate in a study like ours, participants felt it was important to better understand dengue and this would in turn help their community. This seemed to be a satisfactory reason for most to participate. One participant seemed taken aback when probed about her willingness, she replied “*why wouldn’t I participate*!” As described previously, once the FGD participants had their initial concerns and questions addressed and understood the scientific objectives of the study, enthusiasm to participate increased.

Clinical attention that would be received during the study was seen as valuable. FGD participants liked the idea that they would be monitored by a doctor and our research team until they were dengue free. There was interest in knowing how their dengue was progressing and knowing if they were recovering. For example, some expressed interest in knowing if they had anemia, and those who had heard about platelet counts wanted to know if that kind of information would be provided. We clarified that clinical monitoring was not dependent on participating in mosquito feeds or intensive blood draws, because all participants in our studies get medical attention. Most FGD members continued to express willingness to participate. A related motivation was obtaining a dengue diagnosis.

Before we clarified that for our future experiments we would need to directly feed mosquitoes and take tubes of blood, more participants thought feeding mosquitoes directly would more desirable than providing blood samples. It was seen as less painful and would not require as much blood. Some participants also expressed curiosity, stating, “*I want to know how it feels to feed the mosquitoes*”, or “*I would want to see how many mosquitoes got infected if I were sick*”.

### Preference for feeding mosquitoes or providing blood samples

We implemented a variety of exercises to discern people’s attitudes about providing blood samples and feeding mosquitoes, and their perceived advantages and disadvantages for each. These questions were posed after the FG facilitator (VAPS) felt that the participants understood the procedures would only be conducted on DENV-infected people and that the mosquitoes used in the experiments were clean. In the initial FGDs, we asked people which of the two options, direct mosquito feeding or blood collection, they would prefer, followed by an explanation that we would need both and a question about their willingness to provide both. In later FGDs, we started by saying we needed people to feed mosquitoes and give blood samples. We asked whether they would be willing to do both, and the pros and cons of each. About half of participants said they would be willing to do both, whereas a little over a quarter said they would only be willing to feed mosquitoes directly and a little under a quarter said they would only be willing to provide venous blood samples for indirect feeding.

#### Why people preferred feeding mosquitoes over giving blood

For many people, mosquito feeding was preferable to venipuncture. In general, “*fear of needles or venipuncture*” is widespread, and losing blood is a concern for Iquitos residents. Overwhelmingly, respondents that preferred this option felt that mosquito feeding was less painful and was better because they would lose less blood. Others indicated that direct feeds made more sense to them, as that is actually how DENV is spread. There was also a sense that being bitten by mosquitoes was very normal and less scary than a blood draw.

#### Why people preferred venipuncture over direct mosquito feeds

A major reason for choosing to give blood over feed mosquitoes was that this would allowed testing to determine whether they still had dengue, as well as testing for anemia and other clinical indicators. It had more tangible benefits for them. Some in this group also expressed that they felt it was safer, faster, and less likely for them to have a reaction. The perception that blood draws were faster than the 10-minute mosquito feed was an important determinant in this choice. When feeding mosquitoes in their homes was an option, some participants became open to direct feeding because it would reduce the time needed to participate. People who mentioned sensitivity to mosquito bites had a clear preference for providing blood samples and indirect feeding.

It was clear that, as we became more efficient in describing the process (later FGDs), willingness to participate in both direct and indirect feeds increased. When comparing direct feeds to blood draws, we noted a preference for direct feeds over blood draws. Individuals who said they would not participate in either procedure were predominantly older (>60 years of age) individuals who did not grasp the hypothetical nature of our questions. When asked, they would say things like “*but I’m not sick*” or *“[I have to] travel this week*.” These individuals were present in FGDs from both surveillance and non-surveillance areas.

### Participation of children in direct mosquito feeding experiments

Most parents stated that they were comfortable allowing their children to participate in direct feeding if the child could decide. There was some discussion among participants about the age at which children would understand the procedures, but parents felt that, as long as the child’s choice was respected, they would allow their participation. At the same time, many parents were skeptical that their child would choose to participate or be able to sit still throughout the process, with a few FGD participants mentioning how the sight of a nurse caused their children to cry or run away. In contrast, a few parents said that their child would want to participate because they were “*brave*” or that it would be interesting to them. A few people said they would want to experience a feed before allowing their child to participate: “*I want to know what it feels like before giving authorization for my child*.*”* Some parents wanted to ensure that less blood would be taken from children than adults, “*If they take my blood for three days*, *then the child should only do two days…*.” As with adults, some children were recognized as sensitive to mosquito bites; parents of these children would not allow them to participate.

### Participation in multiple direct mosquito feeds and providing multiple blood samples

We used a variety of strategies to probe participants on their willingness to participate in direct mosquito feeding and provide blood samples multiple times. These strategies included asking people to raise their hands and playing games with individuals jumping to the “yes” or “no” side of a line on the floor based on their responses. Responses ranged from none to as many times as necessary (explained by the facilitator as every day they had a fever). Those who answered as often as necessary qualified their responses by stating they would participate “*as long as I still have dengue*,” “*until my platelets are okay*,*”* or “*until my hemoglobin is okay*.” There was a strong connection between willingness to participate and the clinical follow up that would accompany study participation. About 65% of participants who indicated a willingness to participate said they would be willing to repeat the procedures at least three times. Others were willing to have samples taken every other day or at the beginning and end of their illness.

### Acceptability of direct mosquito feeds at home

We asked participants their opinions about conducting direct mosquito feeds in their own homes versus a laboratory setting to determine whether people had safety concerns about performing the experiments in their communities. Overwhelmingly, people preferred their homes. Everyone expressed a desire to participate in the fastest and most convenient procedure. One FGD participant stated a preference for home feeds *“so that my family sees this*.*”* The few individuals who expressed a preference for participating in the procedures in the laboratory gave the following reasons: “*because it is safer and I would be afraid if mosquitoes escaped*, *my family could get sick*,” *“because people in my family would gossip*,” “*I would have more confidence*, *it is more credible…*,” and “*so I can get more information*.” In one FGD, the issue of prying neighbors and their possible negative reactions to mosquito feedings was raised.

### Incentives for study participation

Although we did not include questions about incentives for providing blood samples or participating in direct mosquito feeds in our original FG guide, the issue of incentives emerged during one FG discussion in a group where since 2007, participants received small thank you gifts (i.e., powdered milk, vitamins) after providing blood samples. We asked participants what they thought about incentives and whether they should be given for participation in direct mosquito feeds. One participant stated it would not motivate her, but that “*some people would do the mosquito feeding for money or necessity*.*”* We then asked the group directly whether we should provide incentives, and the group answered, definitively and in unison, “No!” Although none of these participants suggested we should stop providing milk and vitamins for our ongoing studies, there were a variety of strong responses that indicated incentives were not the reason they would participate and that they often had to set their gossiping neighbors straight about this.

### Perceptions from participants in mosquito feeding experiments

#### Focus group composition

All five participants preferred the direct feed to the blood draw, describing it as more comfortable. When asked if they would participate again if they had another DENV infection, all five said they would feed mosquitoes. Two said they did not like giving blood samples. Three of the participants could feel the biting, and although they developed a few small welts from the bites, they reported that these went away quickly. The mother of a 10-year-old participant recounted that her son initially asked to stop the feeding when he could feel the bites, but she asked him to wait and he was able to finish the 10-minute feed. It did not hurt it, just felt strange, he reported. All of these experiments were conducted in the laboratory, but all participants or participants’ parents would have preferred to carry out the feeding procedure in their home.

All of the participants were asked to participate in a mosquito feeding and provide a blood sample only once. If they had dengue another time and were asked to participate, two participants indicated they would participate in both procedures as long as necessary, one said a maximum of three times, and two said they would only participate once while they were ill. We asked what they liked about participation and for a ranking of the following points: attention (clinical examination and care during the procedure), results (complete blood count and laboratory dengue diagnosis), information (about dengue and laboratory and insectary tours), and incentives (drinks and cookies during the feeding procedure, and, during the pilot study, the equivalent of $10.00 cash for time required to participate [adults] or school supplies [children]). Four of five participants ranked attention first; one participant ranked information first. All but one participant ranked incentives last.

#### Experiences and comments from other participants

From September 2012 to January 2016 we enrolled 58 DENV-positive individuals in our pilot study, 53 of which participated in direct mosquito feeds. Two asymptomatic participants were included. Of the participants, 19 were minors (three were 10, five were 12–14, and eleven were 15–17 years of age), the rest were 18–73 years old. Only four of these 58 participants had clearly visible welts where the mosquitoes had bitten them. The rest showed little to no reactions. With the exception of one participant, who was provided an oral anti-histamine, all of these reactions were controlled with itch (betamethasone 5%) cream immediately after application.

Participants described the mosquito biting as a tickle, if they felt them biting at all, with the exception of one 8-year-old girl who asked that the mosquitoes be removed and did not complete the feeding procedure. When the study PI (ACM) was present, she asked six participants after the feed was completed whether they would be willing to participate in additional feeds if the protocol changed to include this in the future. All indicated they would be willing to carry out additional direct feeds if it were necessary for the experiment, but, if it made no difference, they would opt for blood samples because the process was quicker. In fact, many of the early participants expressed concern about the time it took to carry out the procedures and suggested a preference for participating in their homes.

## Discussion

Results from the 12 FGDs, combined with fifty-three direct mosquito feed participants, indicate that mosquito feeding procedures were acceptable to the local Iquitos population, as long as participants were well informed and received a few key messages that were properly reinforced. There were few concerns, either physical and psychological, about discomfort from mosquito bites among FGD participants. Concerns were limited to people with known sensitivity to bites. In contrast, a majority of participants expressed a preference for mosquito feeding over venipuncture. The main complaint about feeding mosquitoes related to the time required (for this pilot study, participants went to the laboratory). Adults supported child participation if the child’s assent was assured, and many were motivated to do so by the need to contribute to “*solving the dengue problem*” and the clinical follow up they would receive as part of such studies. The same concerns and motivating factors were voiced in nearly all of the FGDs, demonstrating saturation [29].

In the FGDs, we used a mosquito feeding video to demonstrate the informed consent process and role-playing exercises and other activities, including demonstrating a mosquito feeding on the study PI, to describe the study. Important community concerns and misconceptions could, however, be effectively addressed with more explanation. We recognize that there are many entomological research tools, such as human-landing collections, mark-release-recapture, insect trapping, and mosquito feeding, that are unfamiliar to most people and may seem unusual or even unethical to those outside the field of mosquito-borne disease [6]. Our proposed direct feeding experiments were no exception and just seemed “*weird*” when first introduced in our FGDs. The initial reactions to the video (in early FGDs, a simple description of the mosquito feeding procedure) were smiles, laughter, and a few squeals. After discussion and clarification, the majority of participants saw the value and reason for the mosquito feeding studies. Because of sensitivity regarding the ethics of these studies, some of the comments and initial misconceptions are of interest and can now be anticipated by our team. Reactions from the FGD participants also illustrate the importance of directly addressing these misconceptions ensuring that every participant has received and understood key messages about the study, including details of the procedures. Communication strategies must be properly assessed, and qualitative research strategies provide a powerful tool to do so.

Over the week we conducted the FGDs, our team adapted from our original goal of assessing acceptability of this kind of study and evaluating the video, to using the video as the primary tool to explain study procedures. It became clear that despite role playing and detailed explanations from our team expert, the video was easier for people to understand. One recommendation from this experience is to develop more audiovisual aids for use in the informed consent process. Limitations are that developing the video was labor intensive, and providing it to our IRB was challenging due to poor internet speed in Iquitos. Informational pamphlets with pictures and captions could be used as an alternative, but it is unclear whether these forms of communication would be as effective as the video, particularly in populations with low literacy.

The most important key messages identified for reinforcement after participants viewed the video are the following: 1) only people infected with DENV would be included in the study, 2) the mosquitoes used in the experiments are reared in our laboratory and DENV-free, 3) each cup of mosquitoes would be used on only one person a single time, and 4) the purpose of direct mosquito feeds is to study what happens to the mosquito, whereas the blood samples help us understand how dengue is affecting the person and may be used to feed mosquitoes in the laboratory. We note that receiving laboratory results was not a requirement for participating in direct feeds. We re-trained our study staff to emphasize each of these messages and have not identified any further misconceptions among our participants or heard rumors about mosquito feeds in the community. In contrast, we had to follow up on periodic rumors about blood samples collected in other projects being pooled for profit or blood transfusions. Our experience is that directly addressing concerns through engagement always reduces concerns and misconceptions.

One key message from these FGDs was the preference for carrying out direct feeds at home. This result was critical for our local IRB and Loreto Regional Health Department to approve home feeds. Although the risks associated with direct mosquito feeds are minimal, the fear of how these studies will be received by the community was a major concern. Our FGD participants discussed the importance of information and that uninformed people could get the wrong idea and become sources of negative gossip. We often heard comments, like “*you have to explain it well*” or “*we understand after you have explained it*, *but not everybody would*.” This is in part related to the idea that being a study subject can have negative connotations in Peru. This view has been promoted by some in the press and regulatory agencies, such as a press release in June 2015 that led to Peru’s National Institute of Health halting all pediatric clinical trials [30] for two years when clinical trial research involving children was reinstated [31].

FGDs with groups who had already participated in research projects with our team indicated that “Proyecto Dengue” had a strong track record of taking care of its participants resulting in trust between researcher and participant. For example, many of these participants felt it was enough for us to explain we were using “*clean*” mosquitoes in our experiments, while one FGD group suggested we make another video or pamphlet describing how the mosquitoes are raised in the insectary and screened for DENV before they are used in experiments. That said, in practice, most of our direct feeding participants seemed satisfied with the video’s explanation, and only a few were curious for more details. The high acceptability of direct feeding observed in FGDs with participants who both had and did not have previous exposure to our research projects suggests that for the issue of direct mosquito feeds our long-standing relationship with certain communities did not significantly affect people’s decisions to participate. Our study illustrates the benefits of formative research to assess acceptability of “unusual” processes in a study population and to identify issues that might be of importance to the population when obtaining informed consent for procedures.

One issue that emerged unexpectedly in our FGDs was the issue of incentives for participating in research. Clinical care and followup was a strong motivator to participate in research studies as was a desire to eventually help the community. Context is important to interpret these responses. Our ongoing epidemiological studies [20, 22, 32] visit cohort members three times per week to identify febrile illness and if reported individuals are offered multiple levels of participation: (1) provide an acute and convalescent blood sample, and receive a clinical evaluation daily until well, or (2) the first option, but if positive, also feed mosquitoes. In direct feeding studies (companion manuscript), about 70% of febrile illness participants indicated a willingness to feed mosquitoes if they were positive, with only 10% expressing any fear of mosquito bites.

Although we did not have permission to carry out direct feeding experiments on participants who were hospitalized, we monitored all of our study participants prospectively throughout the course of their illness, independent of their participation in mosquito feeding, and would recommend that participants present to local hospitals (where care for dengue disease is free of charge in Peru) when clinically justified. Twelve of the 58 participants were hospitalized after participating in the feeding procedures. This would be expected since severe manifestations of dengue disease usually occur at the end of illness and our recruitment strategy was designed to identify dengue cases as early in their infection as possible.

FGDs can provide valuable information to researchers before initiating novel projects. In hindsight, it would have been most useful to carry out the FGDs with people who had never participated in mosquito feeds before initiating our pilot study, and to use the information gained to formulate consent forms and informational materials, including the consent video. Another possible limitation of this study was the differences in when the consent video was viewed in the various FGDs. We found, however, an overwhelming improvement in understanding among those who viewed the video early, and decided to modify the order of events in the field in later FGDs. In previous studies we conducted FGDs before asking participants in a movement study to carry GPS units for 15 days to 3 months [33]. For both mosquito feeds and GPS deployment, anticipated IRB concerns (mosquito bite reactions and privacy concerns, respectively) were of less concern to the participant community than expected. Conversely, we identified unanticipated misconceptions and fears that needed to be addressed directly and clearly.

Our study had some important limitations that impact the generalizability of our results to other parts of the Peru and the World. First, formative research by definition is not intended to provide statistical or completely representative results, rather support or provide context for complementary quantitative studies. Second, our FGs under represented men, because we recruited directly from households during the day, but did so knowing that women are principally responsible for health decisions and childcare. Participation rates in direct feeding experiments [34] suggest that this was not a major bias. Third, Iquitos is a unique community, geographically isolated, with a track record of high community accessibility. We caution generalizing our results to other locations without taking the same steps we did. We also want to send a clear message, that as researchers we need to challenge “perceived” concerns when appropriate but doing so using formative research tools and positive interaction with your IRB to implement needed and research procedures.

### Conclusions

The impact of dengue in Iquitos is large and far-reaching, making people willing collaborators in studies to reduce the local burden of the disease. This, combined with the normalcy of mosquito bites in Iquitos, made direct mosquito feeding experiments, which might seem peculiar to some, acceptable to the majority of our FGD participants, and for parents, also an acceptable experiment to be carried out among children who could assent to the process. Taken in context with the response to direct feeding experiments [34], in Iquitos, when conducted by experienced researchers using a DENV-free generation of mosquitoes raised in a secure insectary, this is a low-risk, non-invasive procedure that is experimentally superior to artificial mosquito feeding methods. The use of formative research, to identify and develop effective communication strategies for “unusual” procedures provides and effective path to opening up new research procedures to communities where they might be appropriate.

## Supporting information

S1 Video Consent video presented to participants at enrollment in mosquito feeding experiments and in focus group discussions corresponding to Fig 1 in manuscript. Video in wnv format.
(WMV)
